# Editorial: Recent advances in Cancer biomarkers detection in biological samples

**DOI:** 10.3389/fchem.2024.1501277

**Published:** 2024-10-09

**Authors:** Francisco G. Ortega, Matías D. Regiart, Martín A. Fernández-Baldo

**Affiliations:** ^1^ Center for Genomics and Oncology Research, Andalusian Autonomous Government of Genomics and Oncological Research (GENYO), Granada, Spain; ^2^ Universidad Nacional de San Luis, Facultad de Química, Bioquímica y Farmacia, Instituto de Química de San Luis, INQUISAL (UNSL - CONICET), San Luis, Argentina

**Keywords:** Cancer biomaker, biosensor, electrochemistry, biological samples, nanomaterials

The identification of biomarkers in biological and clinical samples is a critical component of the emerging paradigm of personalized medicine. With the significant rise in novel targeted therapies and immunotherapies, medical professionals require accurate and reliable biomarker data to make informed decisions regarding the most appropriate treatments for individual patients. Furthermore, the discovery of new biomarkers must be supported by the development of fast, easy, and cost-effective methods to ensure their widespread application in clinical practice.

This Research Topic presents a Research Topic of original articles and reviews that highlight the latest advances in biomarker discovery and detection methodologies.

In their study, Pei et al. conducted a bibliometric and visualization analysis focusing on the use of extracellular vesicles as a liquid biopsy tool for diagnostic biomarkers in osteosarcoma (Pei et al.). Their work sheds light on the potential of circulating as non-invasive diagnostic techniques in cancer care.

Li et al. offered another notable contribution by systematically evaluating the efficacy of one-step nucleic acid amplification (OSNA) for diagnosing sentinel lymph node metastasis in CK19-positive cancers. As sentinel lymph node metastasis is a critical indicator of cancer prognosis, the team conducted a bioinformatic analysis based on data from The Cancer Genome Atlas (TCGA) and the Human Protein Atlas, further underscoring the importance of this method in cancer diagnostics (Li et al.).

Similarly, Liu et al. performed a systematic review and a PRISMA-compliant meta-analysis to assess the diagnostic performance of OSNA in detecting sentinel lymph node metastasis in CK19-positive breast cancer. This comprehensive analysis enhances our understanding of OSNA’s role in cancer diagnosis and patient management (Liu et al.).

Finally, Regiart et al. contributed a systematic review on the latest developments in the electrochemical detection of protein biomarkers in lung cancer. Their review, covering research published in the last 5 years, provides valuable insights into how electrochemical methods can advance cancer biomarker detection, offering a promising avenue for clinical application of their analysis (Regiart et al.).

We extend our sincere gratitude to all the authors and reviewers who have contributed to this Research Topic, as well as the journal’s editorial team for their invaluable support throughout the editorial process.

